# The Bone Morphogenetic Protein Signaling Inhibitor LDN-193189 Enhances Metastasis Development in Mice

**DOI:** 10.3389/fphar.2019.00667

**Published:** 2019-06-19

**Authors:** Julien Vollaire, Irma Machuca-Gayet, Jonathan Lavaud, Aurélie Bellanger, Lamia Bouazza, Soumaya El Moghrabi, Isabelle Treilleux, Jean-Luc Coll, Olivier Peyruchaud, Véronique Josserand, Pascale A. Cohen

**Affiliations:** ^1^INSERM U1209, CNRS UMR5309, Univ. Grenoble Alpes, Institute for Advanced Biosciences, Grenoble, France; ^2^INSERM UMR1033 LYOS, Lyon, France; ^3^University of Lyon 1, Lyon, France; ^4^INSERM U1052, CNRS 5286, Centre de Recherche en Cancérologie de Lyon, Lyon, France; ^5^Department of Biopathology, Centre Léon Bérard, Lyon, France

**Keywords:** breast cancer, ZNF217, bone metastasis, bone morphogenetic protein pathway inhibitor, LDN-193189

## Abstract

Breast cancer with bone metastasis is essentially incurable with current anticancer therapies. The bone morphogenetic protein (BMP) pathway is an attractive therapeutic candidate, as it is involved in the bone turnover and in cancer cell formation and their colonization of distant organs such as the bone. We previously reported that in breast cancer cells, the ZNF217 oncogene drives BMP pathway activation, increases the metastatic growth rate in the bone, and accelerates the development of severe osteolytic lesions in mice. In the present study, we aimed at investigating the impact of the LDN-193189 compound, a potent inhibitor of the BMP type I receptor, on metastasis development *in vivo*. ZNF217-revLuc cells were injected into the left ventricle of nude mice (*n* = 16) while control mice (*n* = 13) were inoculated with control pcDNA6-revLuc cells. Mice from each group were treated or not with LDN-193189 for 35 days. We found that systemic LDN-193189 treatment of mice significantly enhanced metastasis development, by increasing both the number and the size of metastases. In pcDNA6-revLuc-injected mice, LDN-193189 also affected the kinetics of metastasis emergence. Altogether, these data suggest that *in vivo*, LDN-193189 might affect the interaction between breast cancer cells and the bone environment, favoring the emergence and development of multiple metastases. Hence, our report highlights the importance of the choice of drugs and therapeutic strategies used in the management of bone metastases.

## Introduction

Breast cancer is the most frequent cancer among women (Ferlay et al., 2015). More than two-thirds of breast cancer patients are expected to die after the development of bone metastases (BM), primarily osteolytic lesions (Coleman, 2006). Breast cancers with BM are currently mostly incurable; therefore, the identification of suitable therapeutic candidates is of utmost importance.

Previous data strongly suggest that the bone morphogenetic protein (BMP) pathway, a critical regulator of bone homeostasis, may be a promising therapeutic target in tumorigenesis and BM (Bach et al., 2018). Indeed, this pathway is involved in cell-autonomous functions in tumor cells, as well as tumor–stroma interactions in the bone environment (Keller et al., 2001; Barnes et al., 2004; Javed et al., 2005; Alarmo and Kallioniemi, 2010; Sethi and Kang, 2011). The BMP pathway has been ascribed both tumor-promoting or -suppressing activities, according to the context (Bach et al., 2018), though its activation is mainly associated with tumor progression and metastasis development. For instance, in human breast cancer cells, BMP-Smad signaling stimulates development of BM (Katsuno et al., 2008). BMP inhibitors (Jiramongkolchai et al., 2016) thus constitute a promising approach for managing tumorigenesis and breast cancer-derived BM.

We previously reported that ectopic expression of the *ZNF217* oncogene in MDA-MB-231 breast cancer cells leads to the constitutive activation of the BMP pathway, indicating that ZNF217 is a novel upstream BMP signaling activator (Bellanger et al., 2017). A series of *in vitro* experiments showed that BMP signaling is strongly involved in ZNF217-mediated breast cancer cell aggressiveness. Indeed, treatment of MDA-MB-231 breast cancer cells overexpressing ZNF217 with specific BMP inhibitors (one of which being LDN-193189) led to impaired ZNF217-dependent cell migration and cell invasion and impeded chemotaxis to the bone (Bellanger et al., 2017). In mice, experiments conducted by intracardiac injection of ZNF217-positive breast cancer cells revealed that these latter rapidly colonize the bone, leading to the development of severe multiple BM detectable as early as 7 days post-injection (Bellanger et al., 2017). In this well-described and well-characterized in *vivo* model, mice injected with ZNF217-positive breast cancer cells developed osteolytic lesions validated by microCT, and only in extremely rare cases were concomitant metastases at other locations observed (Bellanger et al., 2017). This novel *in vivo* model of BM thus represents an attractive model for testing candidate drugs.

LDN-193189 is a potent inhibitor of the BMP type I receptor (Cuny et al., 2008) and was chosen in our study as its efficacy and toxicity in mice are well characterized (Yu et al., 2008; Boergermann et al., 2010; Lee et al., 2011; Balboni et al., 2013). Furthermore, among the different BMP inhibitors, LDN-193189 has scarcely been tested in the prevention of metastasis development. To our knowledge, only one *in vivo* study reported that LDN-193189 prevents prostate tumor growth rate in the bone and development of osteoblastic lesions (Lee et al., 2011). Based on our novel *in vivo* murine model of osteolytic lesions (Bellanger et al., 2017), we aimed at investigating whether systemic inhibition of the BMP pathway by LDN-193189 could influence metastasis development.

## Materials and Methods

### Cell Culture and Treatments

MDA-MB-231-pcDNA6, MDA-MB-231-ZNF217, and their stable luciferase-transfected derived cell lines pcDNA6-revLuc and ZNF217-revLuc were previously established and described (Thollet et al., 2010; Vendrell et al., 2012). We previously validated in our cell lines the inhibitory action of 10^−7^ M LDN-193189 (Sigma, France) on the BMP pathway (Bellanger et al., 2017). Before injection into mice, pcDNA6-revLuc and ZNF217-revLuc cells were treated for 4 h with 10^−7^ M of LDN-193189 or vehicle (distilled water).

### Animal Models

Experiments were conducted following the European Union guidelines and approved by the ethics committee of Grenoble, France (C2EA-12 ComEth Grenoble). As previously described (Bellanger et al., 2017), pcDNA6-revLuc or ZNF217-revLuc cells (2.5 × 10^5^) were injected into the cardiac left ventricle of *n* = 18 or *n* = 20 6-week-old athymic NMRI nude female mice (Janvier Labs, France), respectively. Cell implantation was immediately controlled by *in vivo* bioluminescence imaging (IVIS Kinetic, PerkinElmer). Only mice, the bioluminescent signal of which was diffused throughout the whole body, were considered to be correctly implanted (13/18 and 16/20, respectively, **Supplementary Figure 1A**) and were included in the following experimental groups: pcDNA6-revLuc (*n* = 5), pcDNA6-revLuc + LDN-193189 (*n* = 8), ZNF217-revLuc (*n* = 8), and ZNF217-revLuc + LDN-193189 (*n* = 8). Subsequently, from day 0 to day 35, pcDNA6-revLuc mice or ZNF217-revLuc mice received daily intra-peritoneal (IP) injections of LDN-193189 (3 mg/kg body weight in distilled water) or vehicle (distilled water). The LDN-193189 experimental setup was based on previous *in vivo* studies (Yu et al., 2008; Lee et al., 2011; Balboni et al., 2013). LDN-193189-treated mice did not exhibit any loss in their body weight, demonstrating that the inhibitor had no severe toxic side effects. Bioluminescence imaging (IVIS Kinetic, Caliper), was performed weekly as previously described (Bellanger et al., 2017). A *p* value of <0.05 was considered statistically significant (Mann–Whitney, StatView™ Software).

**Figure 1:**
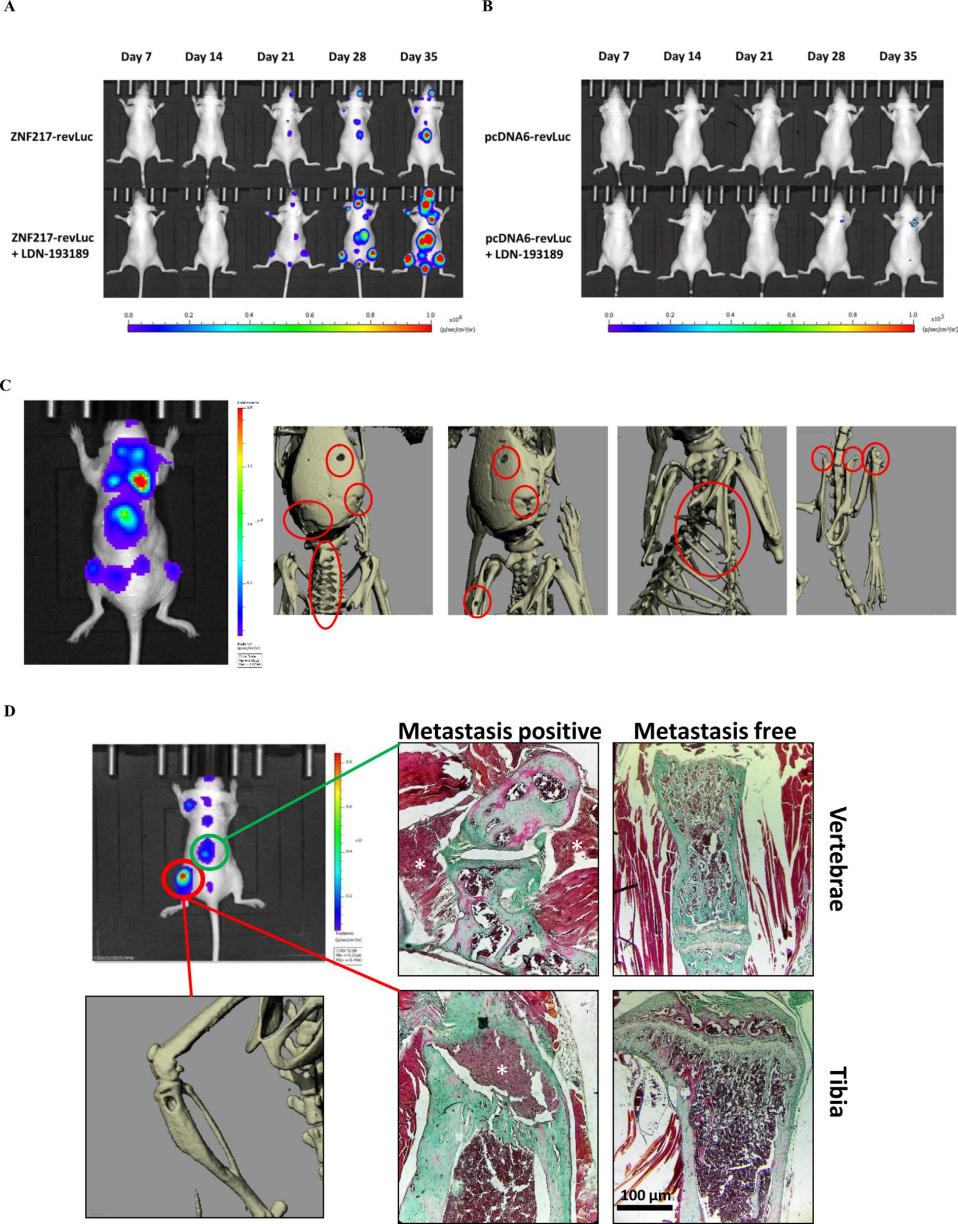
Kinetics and development of metastases *in vivo*. The kinetics of metastases development were followed by bioluminescence in mice injected with **(A)** ZNF217-revLuc cells or **(B)** pcDNA6-revLuc cells, and treated or not with LDN-193189 (representation of one mouse per group representative of the entire group). **(C)** Whole-body bioluminescence and microCT imaging between 35 and 42 days after intracardiac injection of ZNF217-revLuc cells. **(D)** Representative bioluminescence image of an entire mouse exhibiting multiple metastasis foci including an osteolytic BM detected by microCT scan at the left tibia and by histological examination both at the vertebrae and the tibia from bone tissue sections stained with Goldner’s trichrome. Mineralized bone is stained in green and cells are stained in dark red. White stars identify metastasis foci. Histological examination of similar areas from a metastasis-free mouse is also shown in vertebrae and tibia. Scale bars: 100 µm.

### Whole-Body Bioluminescence and X-Ray Microtomography (microCT) Imaging

Five minutes before imaging, vigil mice received an IP injection of 150 µg/g of D-luciferin (Promega) and were then anesthetized (isoflurane 4% for induction and 1.5% thereafter) and placed in the IVIS Kinetic imaging system (PerkinElmer). MicroCT was then performed using the vivaCT40 (ScancoMedical) at 45 keV with a 177-mA intensity, a 200-ms integration time, and an 80-mm isotropic voxel size.

### Histology

Hind limbs and spines from animals were fixed, decalcified in 16% EDTA, and embedded in paraffin. Five-micrometer tissue sections were stained with Goldner’s Trichrome and processed for histological analysis.

## Results

**Figure 1A and B** illustrates the kinetics and amplitude of metastasis development in mice implanted with pcDNA6-revLuc or ZNF217-revLuc cells, treated or not with LDN-193189. The pattern of metastases distribution observed in ZNF217-revLuc cell-injected mice, treated or not with LDN-193189, was totally superimposed with those previously observed in our well-characterized *in vivo* model of osteolytic lesions (Bellanger et al., 2017) (**Supplementary Figure 1B**). MicroCT images are capable of highlighting osteolytic lesions following a longer bone remodeling period, i.e., once the cells had sufficiently colonized the tissue (Sanches et al., 2015). **Figure 1C** illustrates representative microCT images, highlighting osteolytic bone lesions following intracardiac injection of ZNF217-revLuc cells. Histological investigations validated osteolytic lesions and the presence of breast metastatic tumor cells inside and in close contact with bone (**Figure 1D**).

**Figure 2:**
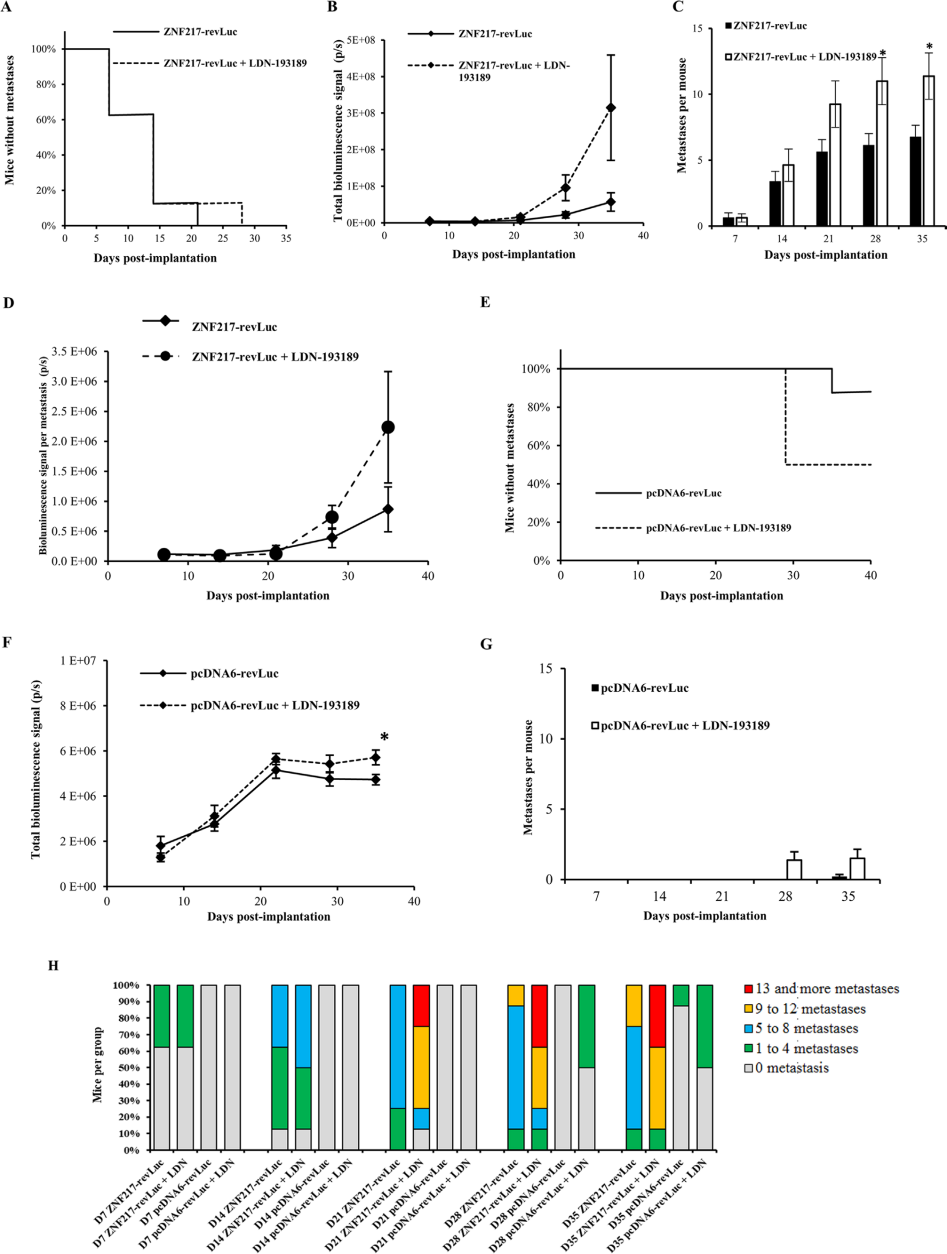
The LDN-193189 BMP inhibitor enhances metastases development *in vivo*. ZNF217-revLuc or pcDNA6-revLuc cells were delivered *via* intracardiac injection into the bloodstream of nude mice. **(A)** Kaplan–Meier analysis of metastasis-free mice in LDN-193189-treated (*n* = 8) and non-treated (*n* = 8) ZNF217-revLuc-injected mice. **(B)** Total metastases load measured by *in vivo* bioluminescence imaging in LDN-193189-treated (*n* = 8) and non-treated (*n* = 8) ZNF217-revLuc-injected mice. **(C)** Average number of metastases per ZNF217-revLuc-injected mouse. **(D)** Total bioluminescent signal per metastasis in LDN-193189-treated and non-treated ZNF217-revLuc-injected mice. **(E)** Kaplan–Meier analysis of metastasis-free mice in LDN-193189-treated (*n* = 8) and non-treated (*n* = 5) pcDNA6-revLuc-injected mice. **(F)** Total metastases load measured by *in vivo* bioluminescence imaging in LDN-193189-treated (*n* = 8) and non-treated (*n* = 5) pcDNA6-revLuc-injected mice. **(G)** Average number of metastases per pcDNA6-revLuc-injected mouse. Results are presented as mean ± standard error of the mean (SEM) in **(B)**, **(C)**, **(D)**, **(F)**, and **(G)**. *P < 0.05 (Mann–Whitney test). **(H)** Distribution of the number of metastases developed by each group of mice.

Intracardiac injection of ZNF217-revLuc cells into mice (*n* = 16) led to the rapid development of multiple bioluminescent metastases (**Figure 2A**). After 7 and 21 days post-injection, 38% and 100% of the untreated injected mice (*n* = 8) developed metastases, respectively, corroborating our previous observation (Bellanger et al., 2017). In the group of ZNF217-revLuc-injected mice treated with LDN-193189 (*n* = 8), the kinetics of metastasis development was very rapid, similarly to that observed in the ZNF217-revLuc-injected mice (*n* = 8) (**Figure 2A**). Strikingly, the total metastases load and the average number of metastases per mouse were higher in the LDN-193189-treated group compared to the non-treated group, and this increase reached significance (*P* = 0.017) at days 28 and 35 post-implantation (**Figure 2B and C**).

All ZNF217-revLuc-injected mice developed multiple metastases, but those who were treated with LDN-193189 displayed much more metastases per mouse compared to the non-treated group (11.0 ± 5.0 vs. 6.1 ± 2.5) at 28 days post-injection (**Figure 2C**). Furthermore, the average bioluminescent signal per metastasis tended to be higher in the LDN-193189-treated group compared to the non-treated group, suggesting that in the presence of this BMP inhibitor, individual metastases are larger (**Figure 2D**).

Unlike ZNF217-revLuc-injected mice, and consistent with our previous report (Bellanger et al., 2017), only 1 out of 5 non-treated pcDNA6-revLuc-injected mice developed a single metastasis, detectable only at 35 days post-injection (**Figure 2E**). The LDN-193189 treatment of pcDNA6-revLuc-injected mice (*n* = 8) affected both the incidence of metastases development (**Figure 2E**), the total metastases load (**Figure 2F**), and the average number of metastases per mouse (**Figure 2G**). Indeed, 50% of LDN-193189-treated pcDNA6-revLuc-injected mice (4 of 8 mice) developed one to four metastases detectable as early as day 28 post-injection, while none were detectable in the non-treated group (**Figure 2E**). Additionally, the bioluminescent signal observed in the LDN-193189-treated pcDNA6-revLuc-injected group, though weaker than ZNF217-induced metastases, was significantly higher than that in the non-treated group at day 35 post-injection (**Figure 2F**, *P* = 0.029). **Figure 2H** illustrates and summarizes the number of bioluminescent metastases in mice injected with pcDNA6-revLuc or ZNF217-revLuc cells, and treated or not with LDN-193189.

## Discussion

Therapeutic BM strategies aiming at targeting the BMP pathway are very attractive, firstly because the latter is involved in the physiology and pathology of bone turnover (Rosen, 2006) and secondly because it might be dysregulated in cancer cells leading to metastases, in particular to the bone (Katsuno et al., 2008). Previous studies reported that i) the DMH1 BMP inhibitor prevents tumor burden in breast cancer and lung metastatic growth (Owens et al., 2015); ii) halofuginone, a dual BMP and TGFβ inhibitor, reduces breast cancer osteolytic lesions (Juarez et al., 2017); and iii) the BMP antagonist noggin prevents development of osteolytic lesions of prostate cancer cells (Feeley et al., 2006).

However, the BMP pathway has paradoxical effects in tumorigenesis and metastasis development, owing possibly to the fine balance between members belonging to this pathway, or between BMP signaling and other pathways, in specific cellular contexts or genetic backgrounds (Jiramongkolchai et al., 2016; Bach et al., 2018). Such complexity is highlighted by apparently conflicting data using BMP inhibitors. Indeed, the BMP antagonist noggin has also been described to contribute to the development of osteolytic BM (Secondini et al., 2011). Moreover, LDN-193189, while inducing decreased tumor burden of colorectal cancer cells (Yokoyama et al., 2017), preventing the growth of pancreatic or breast cancer cells *in vivo*, and increasing survival of mice with ovarian cancer (Lee et al., 2011; Balboni et al., 2013; Ali et al., 2015), was also shown to increase the risk of intestinal carcinogenesis (Whissell et al., 2014).

Using a well-described *in vivo* model of BM metastases in breast cancer (Bellanger et al., 2017), the present report originally investigates the impact of LDN-193189 on metastasis development. Our unexpected and major finding is that systemic treatment with the LDN-193189 molecule has a pro-metastatic effect and stimulates the development of metastases, both in pcDNA6-revLuc-injected mice and in ZNF217-revLuc-injected mice. The impact was significantly greater with ZNF217-positive breast cancer cells displaying aggressiveness and with the ability to develop severe osteolytic lesions. This unexpected result might reflect the impact of this BMP inhibitor on the soil (the bone microenvironment) and/or on the seed (MDA-MB-231 breast cancer cells). Consistently, previous studies suggested that LDN-193189 facilitates bone resorption, represses bone formation, or reduces heterotopic ossification (Yu et al., 2008; Lee et al., 2011; Inubushi et al., 2017). Alternatively, LDN-193189 treatment may induce in both control and ZNF217-postive MDA-MB-231 cells yet uncharacterized molecular events favoring their *in vivo* interaction with the bone environment for osteolytic BM development. Consistently, LDN-193189 displays BMP signaling inhibitory activities on both ZNF217-overexpressing cells and control cells, suggesting that this compound is able to block the activation of both ZNF217-dependent and -independent BMP signaling (Bellanger et al., 2017). Regardless of the context, LDN-193189 treatment seems to accelerate BM development, independently of the level of activation of BMP signaling in breast cancer cells. The concomitant development of metastases to locations other than bone is very rarely obtained in the *in vivo* model used in this study (Bellanger et al., 2017). However, future work investigating whether LDN-193189 treatment impacts metastases development other than in the BM is much needed. Finally, one cannot preclude possible off-target effects of LDN-193189, destabilizing the balance between this pathway and other signaling pathways in the environment or in breast cancer cells (Yu et al., 2008; Vogt et al., 2011).

In conclusion, using an *in vivo* model of breast cancer BM, we found that the LDN-193189 BMP inhibitor displays pro-metastatic properties. Although this study does not refute the use of BMP inhibitors in oncology and in the prevention of metastases, it highlights the necessity to gain further insight into the fine balance governed by the BMP signaling pathway between tumor cells and the environment to improve the development of future drugs.

## Ethics Statement

In vivo experiments (mice) were conducted following the European Union guidelines and approved by the ethics committee of Grenoble, France (C2EA-12 ComEth Grenoble).

## Author Contributions

VJ, J-LC, OP, and PC participated in the design of the study. VJ, JL, and JV performed and analyzed the *in vivo* data. IM-G, LB, SE-M, IT, and OP performed and analyzed the histological experiments. AB participated in previous *in vitro* validation of the LDN-193189 compound and in scientific discussions. JV, AB, VJ, OP, and PC wrote the manuscript.

## Funding

This research program (PAC) was supported by grants from by the French Ligue Contre le Cancer (committees 42 and 71) and Agence Nationale de la Recherche, France (2011 ANR-CESA-018-01). Imaging systems were acquired thanks to France Life Imaging (French program “Investissement d'Avenir” grant; “Infrastructure d'avenir en Biologie Sante”, ANR-11-INBS-0006). OP was supported by grants from INSERM and the University Claude Bernard Lyon-1, the Comité Départemental de la Loire de la Ligue Contre le Cancer, the French Foundation pour la Recherche sur le Cancer (ARC, Grant n°.PJA20151203151), the ANR grant LYSBONE (Grant n°. ANR-15-CE14-0010-01). AB was supported by a PhD grant from the Ligue Nationale Contre le Cancer (LNCC).

## Conflict of Interest Statement

The authors declare that the research was conducted in the absence of any commercial or financial relationships that could be construed as a potential conflict of interest.

## Abbreviations

BM, bone metastases; BMP, Bone Morphogenetic Protein; IP, intra-peritoneal

## Supplementary Material

Figure S1
**(A)** Bioluminescence imaging performed immediately after intracardiac injection of cells expressing luciferase for validation of mice included in the experiments. **(B)** Distribution pattern of the bioluminescent metastases detected in the ZNF217-revLuc injected mice treated with LDN-193189 and ZNF217-revLuc injected mice treated with vehicle.
