# Supplementary figures and images for: The Bone Morphogenetic Protein Signaling Inhibitor LDN-193189 Enhances Metastasis Development in Mice
Source: Front Pharmacol. 2019 Jun 19;10:667. doi: 10.3389/fphar.2019.00667 (PMC6593094; doi:10.3389/fphar.2019.00667)

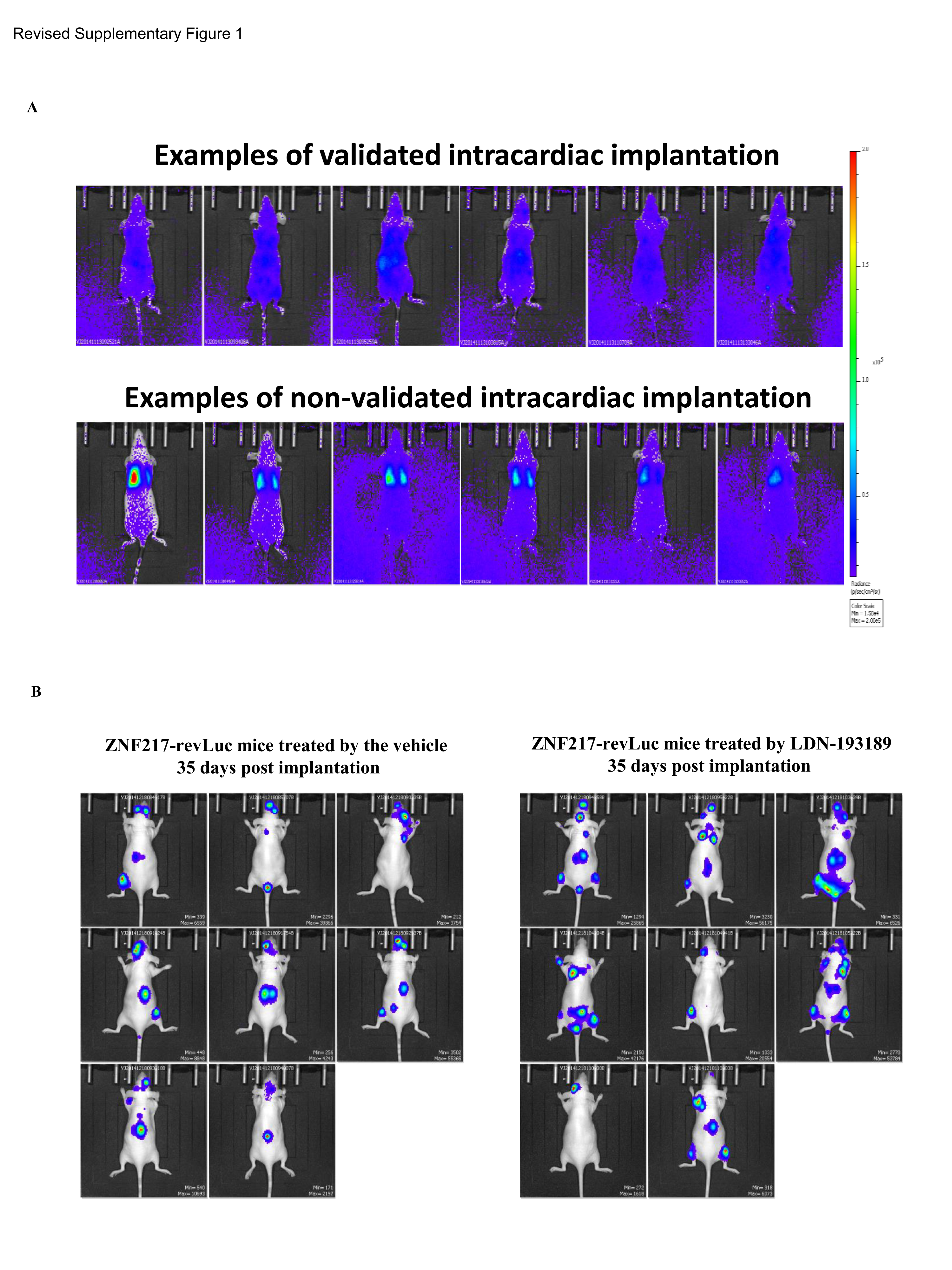

Supplement: Figure S1 — (A) Bioluminescence imaging performed immediately after intracardiac injection of cells expressing luciferase for validation of mice included in the experiments. (B) Distribution pattern of the bioluminescent metastases detected in the ZNF217-revLuc injected mice treated with LDN-193189 and ZNF217-revLuc injected mice treated with vehicle. [file Figure_1.tif]
